# The C Allele of rs5743836 Polymorphism in the Human *TLR9* Promoter Links IL-6 and TLR9 Up-Regulation and Confers Increased B-Cell Proliferation

**DOI:** 10.1371/journal.pone.0028256

**Published:** 2011-11-23

**Authors:** Agostinho Carvalho, Nuno S. Osório, Margarida Saraiva, Cristina Cunha, Agostinho J. Almeida, Maria Teixeira-Coelho, Paula Ludovico, Jorge Pedrosa, Lucia Pitzurra, Franco Aversa, Luigina Romani, António G. Castro, Fernando Rodrigues

**Affiliations:** 1 Life and Health Sciences Research Institute (ICVS), School of Health Sciences, University of Minho, Braga, Portugal; 2 ICVS/3B's - PT Government Associate Laboratory, Braga/Guimarães, Portugal; 3 Microbiology Section, Department of Experimental Medicine and Biochemical Sciences, University of Perugia, Perugia, Italy; 4 Division of Hematology and Clinical Immunology, Department of Clinical and Experimental Medicine, University of Perugia, Perugia, Italy; University of Oklahoma and Oklahoma Medical Research Foundation, United States of America

## Abstract

In humans, allelic variants in Toll-like receptors (TLRs) associate with several pathologies. However, the underlying cellular and molecular mechanisms of this association remain largely unknown. Analysis of the human *TLR9* promoter revealed that the C allele of the rs5743836 polymorphism generates several regulatory sites, including an IL-6-responding element. Here, we show that, in mononuclear cells carrying the TC genotype of rs5743836, IL-6 up-regulates *TLR9* expression, leading to exacerbated cellular responses to CpG, including IL-6 production and B-cell proliferation. Our study uncovers a role for the rs5743836 polymorphism in B-cell biology with implications on TLR9-mediated diseases and on the therapeutic usage of TLR9 agonists/antagonists.

## Introduction

Toll-like receptors (TLRs) are pattern recognition receptors with a major role in activation and homeostasis of the immune system upon pathogen recognition [1]. However, it has become apparent that self-recognition through TLRs can also take place and that this can play a role in sterile inflammation and autoimmunity [2]–[3].

TLR9 activates the innate immune system upon recognition of unmethylated CpG DNA motifs as “danger signals”. In humans, TLR9 is mainly expressed in plasmacytoid dendritic cells and B-lymphocytes [4]–[5], playing a role on viral, fungal, mycobacterial and *Helicobacter pylori* infections [6]–[10] and being implicated in the pathogenesis of several autoimmune diseases [3], [11].

Despite growing evidence of a strong link between deregulated TLR9 responses and immune-mediated diseases, the underlying cellular and molecular mechanisms remain largely unknown. In psoriasis, uncontrolled expression of the antimicrobial peptide LL37 breaks innate immune tolerance by delivering self-DNA to TLR9-containing endosomes, causing TLR9-mediated type I interferon production that drives skin inflammation [3], [11]. Other mechanisms linking TLR9 deregulation and disease may involve TLR9 gain-of-function due to transcriptional deregulation. We and others have shown that the C allele of the single nucleotide polymorphism (SNP) rs5743836 (T-1237C) in *TLR9*, known to display minor allele frequencies ranging from 0.02 to 0.38 across distinct ethnicities, predisposes to non-Hodgkin [12] and Hodgkin lymphoma [13], as well as to several autoimmune and chronic inflammatory diseases, including asthma [14] and Crohn's disease [15].

In light of TLR9 relevance in disease, synthetic oligodeoxynucleotides that specifically inhibit or activate TLR9 have been developed [16]. These compounds can potentially be used in infectious diseases, allergy/asthma and cancer therapy to either block TLR9 self-recognition or stimulate the immune response in conditions of immune tolerance. Given the highly promising effects of CpG-based therapy, understanding the biological and functional impact of *TLR9* genetic variability is pertinent as it will certainly contribute to anticipate potential side-effects associated with TLR9 agonist/antagonist therapy.

Here, we report that the C allele of rs5743836 introduced a new IL-6-dependent transcription factor binding site in the TLR9 promoter. Peripheral blood mononuclear cells (PBMCs) harboring the TC genotype of rs5743836 show higher expression of both TLR9 and IL-6 and increased B-cell proliferation in response to CpG, functionally dependent on IL-6 signaling. Our findings not only contribute to a better understanding of the functional impact of *TLR9* overexpression, but also raise important questions on the therapeutic usage of CpGs in the context of the immunogenetic profile of the patient.

## Results and Discussion

### The C allele of rs5743836 introduces a functional IL-6-responsive element in the *TLR9* promoter

**Figure 1:**
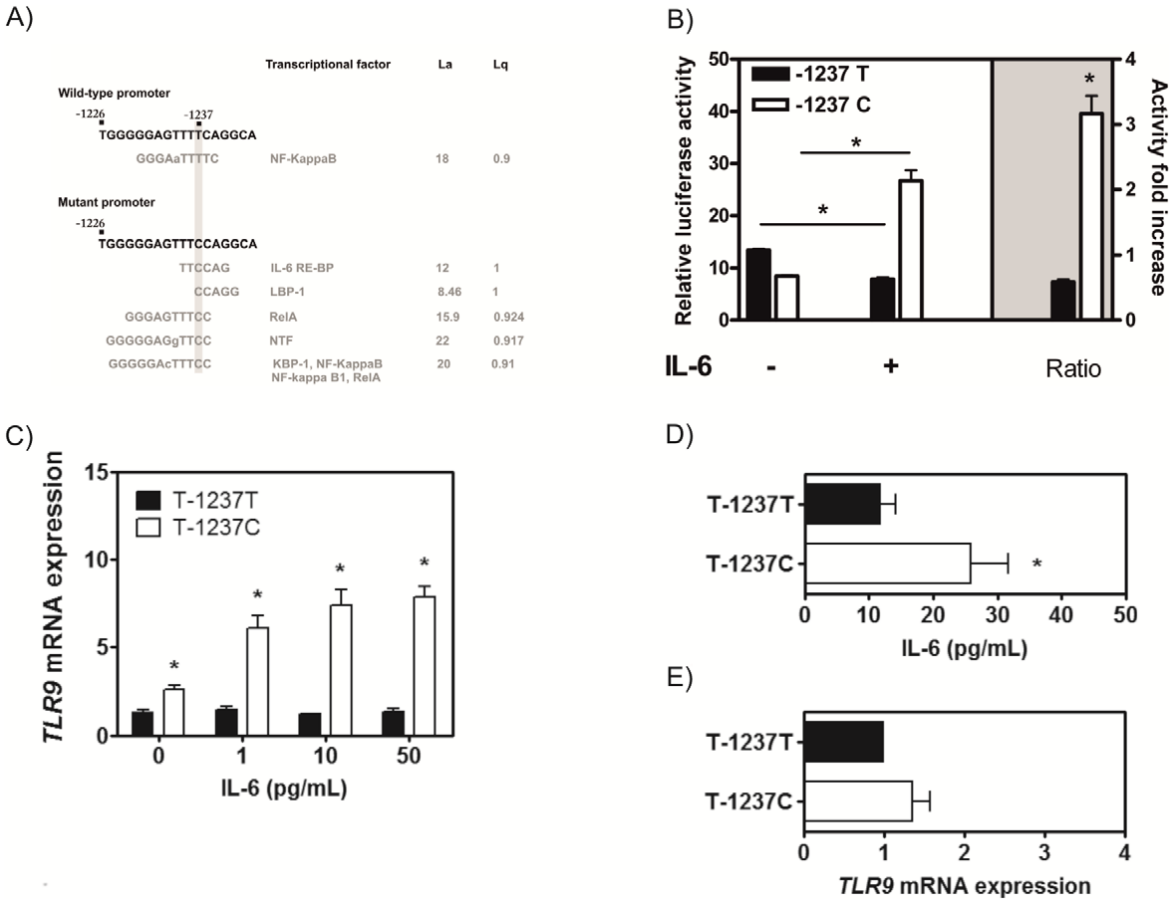
IL-6 increases the activity of the *TLR9* promoter carrying the C allele of rs5743836. (A) *In silico* analysis of the fragment of the *TLR9* promoter containing the T/C substitution using the TESS interface. L_a_, log-likelihood score, L_q_, a measure of the goodness-of-fit of the DNA sequence to the consensus binding motif. (B) Luciferase reporter assay of Raji B cells transfected with plasmid vectors containing the luciferase gene under the control of a 3.2 kb fragment of the promoter sequence carrying the T or C allele. After transfection, cells were left untreated or stimulated with IL-6 (n = 3). (C) IL-6 dose-dependent stimulation of either TT (n = 27) or TC (n = 25) PBMCs. Cells were left untreated or stimulated with increasing doses of recombinant IL-6. *TLR9* mRNA expression levels were determined by real-time PCR. (D) IL-6 secretion by of either TT (n = 21) or TC (n = 17) PBMCs. IL-6 in untreated cells was quantified by ELISA. (E) *TLR9* expression in TT (n = 21) or TC (n = 17) PBMCs in IL-6-neutralizing conditions. Cells were cultured with an anti-IL-6 antibody and *TLR9* mRNA expression was determined by real-time PCR. Results shown are the mean ± SD.

Several SNPs in *TLR9* have been associated with increased risk for immune-mediated diseases (International HapMap Project; http://hapmap.ncbi.nlm.nih.gov/). We hypothesized that some of these SNPs might alter the transcriptional regulation of *TLR9*, leading to inappropriate gain- or loss-of-function. To test this, we screened the transcription factor-binding profile of the human *TLR9* promoter for alterations introduced by known SNPs. *In silico* analysis revealed that the C allele of rs5743836 generated several novel binding sites for different transcription factors (Fig. 1A). The potential regulatory transcription factor-binding motif with the highest score corresponded to an interleukin-6 (IL-6) response element (IL-6 RE) at position -1238 to -1234 with the consensus sequence TTCCAG (Fig. 1A). This type II IL-6 consensus motif has already been shown to play a key role on STAT3 transactivation of the human γ-fibrinogen promoter triggering up-regulation [17], but was never studied in the context of TLR9.

To investigate whether the TLR9 promoter carrying the C allele of rs5743836 was regulated by IL-6, we measured the ectopic expression of luciferase under control of the TLR9 promoter sequence with either the T or C allele in transiently transfected B-cells. As shown in Fig. 1B, upon stimulation with recombinant human IL-6, cells carrying the variant construct displayed a significant 3.2-fold increase in luciferase activity (p<0.001), whereas this effect was not observed for the wild-type construct. This result indicates that rs5743836 regulates the transcriptional activity of the *TLR9* promoter in an IL-6-dependent manner.

We next evaluated whether the C allele of rs5743836 in its native chromosomal context also led to an IL-6-dependent increase in *TLR9* mRNA expression. For this, PBMCs with the TT or TC genotypes were stimulated with increasing doses of recombinant IL-6. We observed that only TC PBMCs responded to the presence of IL-6 with increased *TLR9* expression, in a dose-dependent manner (Fig. 1C).

In addition, we observed that, as compared to TT, unstimulated TC PBMCs displayed an approximately 2.4-fold increase in the basal transcription of *TLR9* (p = 0.008) (Fig. 1C), due to basal production of IL-6 (Fig. 1D). Interestingly, the neutralization of endogenous IL-6 resulted in a decreased *TLR9* expression to levels similar to those of TT cells (Fig. 1E). Overall, our data point towards a major impact of the rs5743836 polymorphism on the transcriptional regulation of *TLR9* and support an IL-6-dependent modulation of *TLR9* gene expression.

### B cells harboring the TC genotype of rs5743836 show increased proliferation upon CpG stimulation

**Figure 2:**
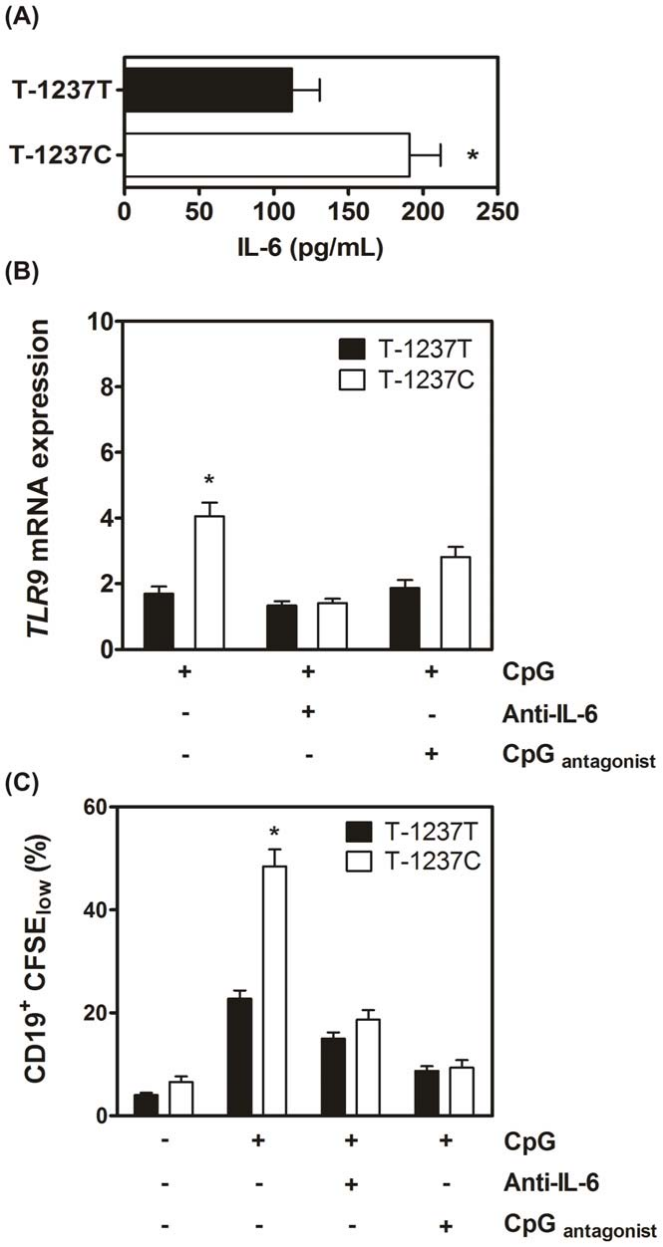
Human PBMCs carrying the TC genotype of rs5743836 stimulated via TLR9 show increased transcription of *TLR9* and enhanced B-cell proliferation. (A) TC PBMCs secrete higher amounts of IL-6 upon activation of TLR9 with CpG ODN 2006. IL-6 production was quantified by ELISA in the supernatants of TT (n = 22) or TC (n = 17) PBMCs following culture with CpG. (B) TLR9 activation of TC cells by CpG ODN 2006 increases *TLR9* gene expression. Quantification of *TLR9* mRNA expression in either TT (n = 24) or TC (n = 27) PBMCs cultured with: CpG ODN 2006 (0.05 µM); CpG ODN 2006 and anti-IL-6 antibody; CpG ODN 2006 and the TLR9 antagonist CpG ODN TTAGGG in a 1∶2 ratio. (C) Proliferation of CD19^+^ cells was assessed by CFSE dilution in PBMCs with different rs5743836 genotypes, TT (n = 40) and TC (n = 33), left untreated or stimulated with: CpG ODN 2006 (0.05 µM); CpG ODN 2006 and anti-IL-6 antibody; CpG ODN 2006 and CpG ODN TTAGGG in a 1∶2 ratio. Results shown are the means ± SD.

To investigate whether the transcriptional upregulation of *TLR9* caused by the TC genotype of rs5743836 led to a gain-of-function phenotype, TC PBMCs were stimulated with CpG. Although IL-6 was produced upon TLR9 activation regardless of the genotype, its production was higher in TC cells (p = 0.009) (Fig. 2A). We next questioned whether in TC cells, CpG stimulation, via IL-6 production, could lead to further up-regulation of *TLR9* transcription and thus to a perpetuation loop of TLR9-mediated mechanisms. We stimulated PBMCs with CpG and measured *TLR9* transcription. We observed that CpG induced a strong increase in *TLR9* expression in TC (p<0.0001), but not TT cells (Fig. 2B). This up-regulation of *TLR9* expression was abrogated by neutralizing the biological activity of IL-6 (Fig. 2B). In the presence of a *TLR9* antagonist (TTAGGG), the up-regulation of *TLR9* expression upon CpG stimulation was only partially reverted (Fig. 2B), probably due to the basal presence of IL-6 (Fig. 1C and 1D). Altogether, our data suggest that PBMCs carrying the TC genotype of rs5743836 are more responsive to CpG stimulation and that this effect is most likely perpetuated by increased *TLR9* transcription and IL-6 production.

Taking into consideration that B-cells constitute the major cellular population expressing *TLR9* within PBMCs (4), and given that rs5743836 affected the levels of both *TLR9* and IL-6 in response to CpG, we investigated whether the presence of this variant impacted B-cell proliferation. To answer this question, PBMCs were stimulated with CpG, and CD19^+^ cell proliferation was assessed. CD19^+^ cell proliferation induced by CpG was significantly higher in TC cells as compared to TT cells (48.5% vs. 22.8%, respectively; p<0.0001) and was abrogated by co-incubation with a *TLR9* antagonist (Fig. 2C). In line with the fact that *TLR9* signaling is stronger in cells overexpressing *TLR9* [4], [18], and that IL-6 mediates TLR9 up-regulation in TC cells, neutralization of IL-6 abrogated the increased proliferation of these cells in response to CpG to levels similar to those of wild-type cells (Fig. 2C). Again, our data support an amplification loop mediated by CpG and IL-6 that culminates with a deregulated TLR9 signaling.

**Figure 3:**
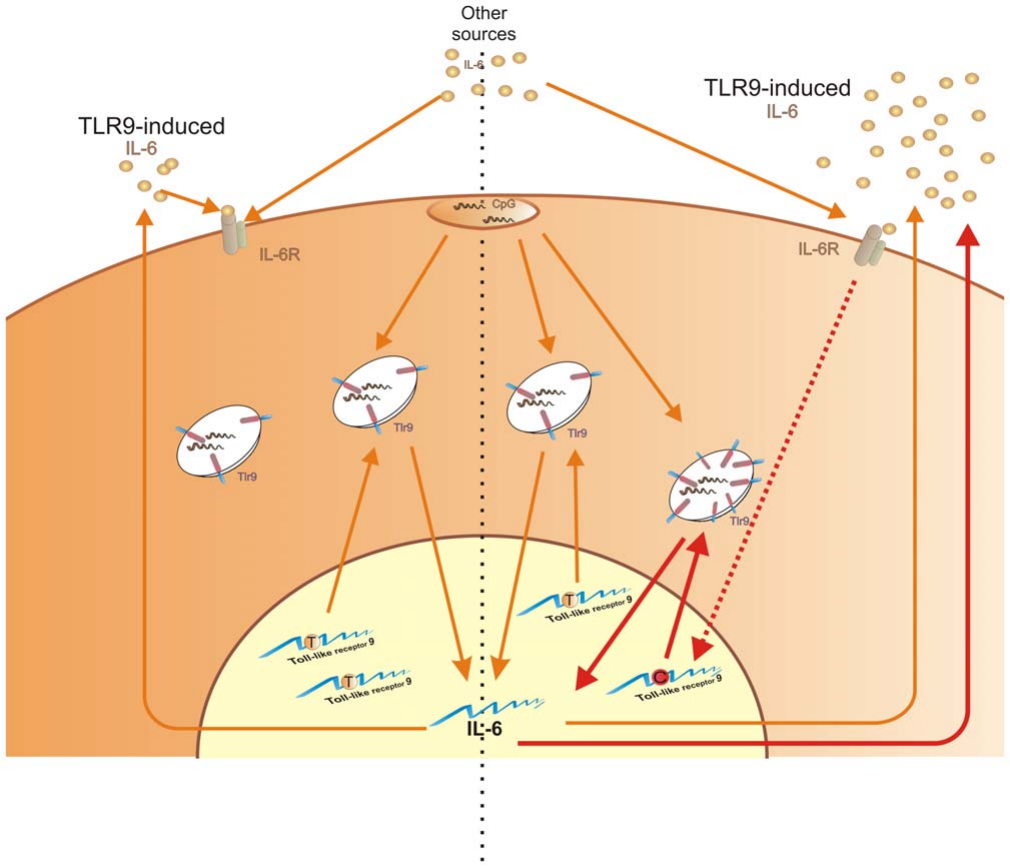
Proposed model of the effect of rs5743836 in B-lymphocyte activation and proliferation. TLR9 activation by CpG DNA induces the production of IL-6, which upon binding to its receptor, promotes translocation of STAT3 to the nucleus. This transcription factor can bind to and trans-activate promoters containing STAT3 binding elements, thus initiating a specific transcriptional program. In cells harboring the TC genotype of rs5743836, STAT3 will bind to a new IL-6 RE site, created by the T/C substitution within the *TLR9* promoter. As a result, *TLR9* expression increases in response to IL-6. Therefore, in these cells, a loop of TLR9/IL-6 signaling amplification is created, leading to a deregulation in B-cell activation and proliferation upon CpG stimuli. Of note, the IL-6 needed to generate this loop may be TLR9-independent.

Altogether, our data indicate that TLR9 signaling is amplified by a positive feedback loop through IL-6 in TC carriers of rs5743836 (Fig. 3), an example on how functional genomic studies may give insights on the regulation of gene activity under different situations, and can contribute to our knowledge on molecular and genetic mechanisms underlying disease susceptibility. In summary, we present evidences that shed light on how a certain genetic profile associated with excessive immune/inflammatory activation may contribute to human disease. Our findings not only identify a new regulatory element in the promoter region of *TLR9*, leading to its transcriptional activation through the IL-6 signaling pathway, with implications on cellular physiology, but also demonstrate the possibility to abolish the cellular effects of this SNP thus strongly supporting new potential individually-targeted treatments. In addition to the broad implications of our study in immune-mediated diseases, another important issue raised by our data is related to the therapeutic application of CpG-based compounds. As we now show, individual genetic variations can affect the outcome of the TLR9 signalling pathway. Re-interpreting CpG therapy trial results, taking into consideration the genotype of the participants, may therefore be of interest, since the global results may have been masked by the presence of the relatively common rs5743836 SNP. Ultimately, the therapeutic applications of CpG should be individually tailored as they may prove to be either detrimental or more effective for individuals harbouring the TC genotype of rs5743836.

## Materials and Methods

### Ethics Statement

Study protocols were approved by Instituto Português de Oncologia de Lisboa, Francisco Gentil, EPE and Instituto Português de Oncologia de Coimbra, Francisco Gentil, EPE, Portugal. All study participants provided written informed consent prior to biospecimen collection.

### In silico analysis of TLR9 promoter allelic variants

Allelic variants within *TLR9* promoter were analyzed to predict alterations in transcription factor-binding sites, using the TESS interface (http://www.cbil.upenn.edu/cgi-bin/tess/). Parameters used in motif prediction included L_a_, log-likelihood score, and L_q_, measure of the goodness-of-fit of the DNA sequence to the consensus binding motif; the best possible L_q_ value was 1.000.

### Plasmid constructs and mutagenesis

A plasmid vector containing the luciferase gene under control of the wild-type *TLR9* promoter [19] was used for site-directed PCR mutagenesis. Briefly, the substitution at position -1237 was introduced by all-round PCR amplification using the primers 5′-TATGAGACTTGGGGGAGTTTC CAGGCAGAGGGAACAGCACA-3′ and 5′-TGTGCTGTTCCCTCTGCCTGGAAACTCCCCCAAGTCTCATA-3′. The correct construct was confirmed by direct sequencing.

### Cell lines and cell culture

The Raji B-cell line (ATCC; wild-type for rs5743836) were cultivated in RPMI culture medium with 2 mM L-glutamine, 1.5 g/L sodium bicarbonate, 4.5 g/L glucose, 10 mM HEPES, 1.0 mM sodium pyruvate and 10% fetal bovine serum - FBS] without antibiotics.

### Transient transfection and luciferase reporter gene assay

Raji cells (5×10^6^ cells/ml) were transfected using the Microporator system (NanoEnTek Inc, Korea) following manufacturer instructions. Transfection efficiency was normalized by co-transfection of 0.5 µg of the control β-galactosidase vector (pCMV-βgal). After recovery, human recombinant IL-6 (100 ng/ µl) was added to 1×10^6^ cells for 12 hours. Cells were lysed with 1× Passive Lysis Buffer (Promega, USA) and luciferase activity was measured using the Luciferase Assay System (Promega). Firefly, luciferase activity was normalized against β-galactosidase activity, determined with the β-galactosidase Enzyme Assay System (Promega). Data are expressed as relative luciferase units.

### PBMC culture

PBMCs of healthy blood donors were isolated using Histopaque-1077 (Sigma, USA). Given the very low frequency of individuals with the CC genotype, PBMCs with TT and TC genotypes were used throughout. Cell viability was determined using trypan blue exclusion. 1×10^6^ PBMC/ml were seeded in 24-well flat bottom plates and cultivated for 5 days with 0.05 µM CpG ODN 2006 (5′-TCGTCGTTTTGTCGTTTTGTCGTT-3′) (InvivoGen, France). Cells either left unstimulated or stimulated with control CpG ODN 2006 (5′-TGCTGCTTTTGTGCTTTTGTGCTT-3′) (InvivoGen) were included as controls throughout the study.

### CFSE proliferation assay

PBMCs (2.5×10^6^ cells/ml) were resuspended in serum-free Hank's Balanced Salt Solution (HBSS) and labeled with CFSE (Molecular Probes, USA) at a concentration of 5 µM. Labeling was quenched by the addition of 1/5 of the total volume of FBS. After washing with complete RPMI medium, cells (2×10^6^ cells/ml) were cultured in 24-well plates for 5 days in medium alone or with CpG ODN plus or minus rabbit anti-IL-6 antibody (Sigma-.Aldrich) or CpG antagonist. Cells were washed, resuspended in staining buffer (0.5% BSA, 0.04% EDTA, 0.05% sodium azide in PBS) and stained with APC-conjugated anti-human CD19 (Becton Dickinson, San Jose, CA, USA) and analyzed on a BD FACSCalibur flow cytometer (Becton Dickinson).

### Real-time RT-PCR

Total RNA was isolated using the RNeasy Mini Kit (Qiagen, Germany) and reverse-transcribed using the iScript cDNA Synthesis Kit (Bio-Rad, France). For RT-PCR, 1 µl of cDNA was used as template using the QuantiTect Sybr Green PCR Kit (Qiagen). RT-PCR primers were as follows: β-actin, sense: 5′-GCCGTCTTCCCCTCCATCGTG-3′, antisense: 5′-GGAGCCACACGCAGCTCATTGTAGA-3′; TLR9, sense: 5′-CAGCAGCTCTGCAGTACGTC-3′, antisense: 5′-CTCAGGCCTTGGAAGA AGTG-3′. RT-PCR was conducted on a LightCycler System (Roche Applied Science, Switzerland) and the thermal cycling conditions included 50 cycles of 10 s at 95 °C, 20 s at 55 °C, and 20 s at 72 °C, after a 15-min initial step of enzyme activation at 95 °C, a melting step of 55–95 °C (0.5 °C/s) and a final cooling step at 40 °C. Expression of β-actin was used as internal control. Expression of *TLR9* is indicated as n-fold increase relative to the level of *TLR9* expression in untreated wild-type cells, using the 2^-ΔΔCt^ method.

### IL-6 quantification

IL-6 was quantified in supernatants from PBMC cultures either in untreated conditions or stimulated for 5 days with CpG ODN 2006 using human IL-6 Quantikine ELISA kit (R&D Systems, Minneapolis, MN, USA).

### Statistical analysis

Data were analyzed by GraphPad Prism 4.03 program (USA). Unpaired Student's T-test with Bonferroni's adjustment was used to determine statistical significance (p<0.05). All experiments were performed at least in triplicate and repeated at least twice.
